# MALDI mass spectrometry imaging (MSI) reveals molecular and structural heterogeneity of amyloid-β in sporadic Alzheimer’s disease and Down syndrome

**DOI:** 10.1186/s40478-026-02280-4

**Published:** 2026-04-02

**Authors:** Karolina Minta, Linda Söderberg, Eleni Gkanatsiou, Malin Johannesson, Christer Möller, My Björklund, Gunilla Osswald, Lars Lannfelt, Susanne Fabre, Wojciech Michno

**Affiliations:** 1 BioArctic AB, Warfvinges Väg 35, 112 51 Stockholm, Sweden; 2 Department of Public Health and Caring Sciences, Molecular Geriatrics, Uppsala University, 75237 Uppsala, Sweden; 3 Science for Life Laboratory, Uppsala University, 752 37 Uppsala, Sweden

**Keywords:** Alzheimer’s disease, Amyloid β, Down syndrome, Matrix-assisted laser desorption/ionization-mass spectrometry imaging

## Abstract

Alzheimer’s disease (AD) and Down syndrome (DS) are both characterized by early accumulation of amyloid-β (Aβ), but the underlying mechanisms differ. In DS, lifelong overproduction of Aβ due to triplication of the APP gene drives pathology, whereas in sporadic AD (sAD) impaired clearance and altered processing are considered to be major contributors to Aβ pathology. Despite these shared hallmarks, it remains unclear whether the molecular composition of plaques, such as Aβ isoform distribution and post-translational modifications, is truly comparable between the two conditions. Most published studies rely on bulk tissue or antibody-based methods, which average across plaques and overlook truncated or modified Aβ isoforms, such as N-terminally or C-terminally truncated forms or pyroglutamate-modified species. Here, we applied a reflector-mode matrix-assisted laser desorption/ionization mass spectrometry imaging (MALDI-MSI) approach, integrated with histology and immunoassay, to characterize Aβ pathology in postmortem brain tissue from DS, sAD, and non-demented control patients at single-plaque resolution. We demonstrate that Aβ plaques and neurofibrillary tangles are significantly larger in DS than in sAD, consistent with more aggressive disease progression. Molecular profiling revealed distinct peptide repertoires between the diseases. DS plaques contained nearly twice as many N-terminally truncated Aβ peptides as sAD, with proportionally similar contributions from Aβx-40 and Aβx-42, and uniquely harbored Aβ2-42, AβpE3-42, Aβ3-40, Aβ4-42, Aβ8-42, and AβpE11-40/42. In contrast, sAD plaques were dominated by truncated Aβ40 while Aβ42 remained largely full-length, and only sAD contained bi-terminally truncated isoforms such as Aβ2-37, Aβ2-39, and Aβ9-38. Overall, inter-peptide correlations between the relative abundances of all Aβ peptides as performed across individual plaques were significantly stronger in sAD than in DS, indicating that peptide levels co-vary more consistently within sAD plaques. Collectively, these findings indicate that Aβ plaques in DS and sAD differ in their molecular composition and peptide profiles. Beyond providing mechanistic insight, these findings highlight the need to tailor Aβ-targeting therapies to disease-specific peptide signatures, particularly for individuals with DS, who are at exceptionally high risk of dementia and have been underrepresented in clinical research.

**Supplementary Information:**

The online version contains supplementary material available at 10.1186/s40478-026-02280-4.

## Introduction

Alzheimer’s disease (AD), a progressive neurodegenerative disorder, and Down syndrome (DS), a genetic condition caused by trisomy 21, both feature early accumulation of amyloid-β (Aβ)[1]. Although the two disorders share this hallmark pathology, they arise from distinct mechanisms. In AD, impaired Aβ clearance and altered processing occur [2], whereas in DS, lifelong Aβ overproduction results from the triplication of the amyloid precursor protein (APP) gene [3]. By midlife, virtually all individuals with DS harbor Aβ plaques, and most develop dementia a decade or two earlier than those with sporadic AD (sAD) [4]. This predictable and aggressive trajectory makes DS a uniquely informative clinical population for understanding the biology of Aβ deposition. At the same time, individuals with DS remain overlooked in therapeutic development, despite being among those most affected by Aβ pathology. The recent approval of Aβ-targeting antibodies highlights this gap: while such therapies are being evaluated for sporadic and familial AD, their efficacy and safety in DS, who exhibit distinct Aβ peptide profiles, remain almost entirely unexplored [5, 6]. A deeper molecular understanding of Aβ pathology in DS is therefore urgently needed, both to illuminate disease-specific mechanisms, and to ensure that individuals with DS are not excluded from the benefits of emerging treatments.

Aβ peptides are generated by sequential proteolysis of APP, yielding a spectrum of isoforms that differ in length and aggregation properties [7]. Among these, Aβ1-40 and Aβ1-42 are the most abundant, with Aβ42 being particularly prone to oligomerization and protofibril formation [8]. In addition to these full-length forms, truncated variants arise through further processing by proteases such as BACE1 (which acts both as a β-secretase initiating Aβ production and as an Aβ-degrading enzyme (ADE) generating N-terminally truncated species such as Aβ pE3-42 and Aβ4-42), BACE2, aminopeptidases, and γ-secretase trimming, as well as through the activity of other ADEs, including neprilysin and insulin-degrading enzyme (IDE). These enzymes contribute to the heterogeneity of Aβ peptides observed in the human brain by generating N-terminal (e.g., Aβ3-x, Aβ11-x) and C-terminal (e.g., Aβ1-37, Aβ1-38, Aβ1-39) truncated species, which can profoundly alter aggregation kinetics, seeding efficiency, and toxicity [9–12]. Soluble Aβ oligomers and protofibrils are thought to be the most neurotoxic forms, and thus primary targets for therapeutic intervention [13]. The balance of full-length versus truncated peptides may therefore critically shape disease trajectories in AD and DS.

Previous comparative studies of DS and AD have reported both similarities and differences in the composition of Aβ pathology. PET imaging and bulk analyses of homogenized brain tissue have shown comparable rates of accumulation and overlapping peptide profiles [12, 14–19], whereas other work suggests earlier deposition and higher absolute levels of Aβ40 and Aβ42 in DS [20]. A major limitation of these approaches, however, is that they average across many plaques and often rely on antibody-based detection, capturing only selected epitopes. As a result, they obscure the molecular heterogeneity of individual lesions and overlook truncated or modified isoforms not recognized by available antibodies.

It is increasingly recognized that Aβ plaques are not chemically uniform but rather mosaics of distinct Aβ isoforms arranged in spatially distinct microdomains, with compositions and post-translational modifications that vary with plaque type, maturation state, and disease progression [21]. These patterns likely reflect specific aggregation pathways and proteolytic environments and resolving them at the level of single plaques is critical for understanding disease-specific mechanisms. Matrix-assisted laser desorption/ionization mass spectrometry imaging (MALDI-MSI) provides precisely this capability, enabling direct, label-free detection of peptides within intact tissue sections while preserving spatial context. Prior work in AD done by us, and others, has shown that MALDI-MSI can resolve heterogeneous Aβ peptide repertoires across plaques [22–30], but this approach has not yet been systematically applied to DS.

The APOE genotype is a major genetic risk factor for sAD, influencing Aβ aggregation, deposition, and clearance. Specifically, the APOE ε4 allele is associated with increased Aβ plaque burden and accelerated aggregation compared to ε3 and ε2 alleles [31]. The isoform-dependent pattern of Aβ accumulation is primarily modulated via differential regulation of Aβ clearance from the brain [32].

In this study, we employed a newly optimized reflector mode MALDI-MSI approach, combined with fluorescence histology and immunoassay, to characterize the composition and spatial organization of Aβ peptides in postmortem brain tissue from individuals with DS, sAD, and age-matched non-demented controls. By interrogating Aβ pathology at the level of individual plaques, we aimed to uncover disease-specific peptide signatures and aggregation patterns that would be masked in bulk methods. Our findings reveal marked differences in the truncation profiles, spatial organization, and peptide co-localization of Aβ in DS and sAD, highlighting distinct disease-specific mechanisms underlying Aβ generation, processing, aggregation, and plaque deposition in each condition. These insights are not only mechanistically informative but also clinically relevant, suggesting that therapeutic strategies targeting Aβ may need to be tailored to the molecular signatures that distinguish DS from sAD.

## Materials and methods

### Patient selection and characteristics

Post-mortem human brain tissue was obtained from the Netherlands Brain Bank (NBB), Netherlands Institute for Neuroscience, Amsterdam (see Table 1 for demographic characteristics). Brain tissues from sAD (n = 3) and DS (n = 3) patients were selected to be matched based on Braak stage (≥ 5), APOE genotype (ε3/ ε3), and gender (1:2, male:female). Only APOE ε3/ε3 individuals were included to minimize confounding effects of the ε4 allele, which can directly influence amyloid deposition, and to allow a clearer assessment of Aβ processing and pathology development independent of APOE genotype. A non-demented control group (n = 3) with low pathology (Braak stage ≤ 2) was also included. All samples were obtained from the same brain region—the frontal gyrus. All material has been collected from donors whose written informed consent for brain autopsy and the use of the material and clinical information for research purposed has been obtained by the NBB. The informed consent form of the NBB meets all current legal and ethical requirements for brain autopsy, tissue storage and use of tissue and clinical data for scientific research worldwide. The study followed the Helsinki Declaration and was approved by the Swedish Ethical Review Authority (2020-00527 and 2021-00965).

**Table 1:** Patients’ demographics

Diagnostic group	ID	Age	Gender	APOE genotype	Braak stage	Brain region
Down syndrome	DS1	58	F	3/3	VI	Frontal gyrus
	DS2	67	F	3/3	VI	Frontal gyrus
	DS3	64	M	3/3	V	Frontal gyrus
Alzheimer’s disease	sAD1	68	F	3/3	V	Frontal gyrus
	sAD2	79	F	3/3	V	Frontal gyrus
	sAD3	56	M	3/3	VI	Frontal gyrus
Non-demented controls	Ctr1	79	M	3/3	II	Frontal gyrus
	Ctr2	73	F	4/4	I	Frontal gyrus
	Ctr3	80	F	3/4	I	Frontal gyrus

### APOE genotyping

APOE genotyping for non-demented control samples was either provided by the NBB or determined using a commercial qPCR kit (Creative Biogene, Shirley, NY, USA). Genomic DNA was extracted prior to analysis using the QIAwave DNA Blood & Tissue Kit (Qiagen, Hilden, Germany).

### Chemicals and reagents

All chemicals for matrix and solvent preparation were pro-analysis grade and obtained from Sigma-Aldrich/Merck (St. Louis, MO, USA) unless otherwise specified. Deionized water was obtained by a Milli-Q purification system (Millipore Corporation, Merck, Darmstadt, Germany).

### Tissue sectioning

For correlative MSI/GeoMx analyses, sagittal cryosections (10 μm) were prepared from fresh-frozen brain tissue using a cryostat microtome (Leica CM1520, Leica Biosystems, Nussloch, Germany) operated at − 18 °C. Sections designated for GeoMx DSP were thaw-mounted onto SuperFrost Plus slides, while adjacent sections were placed on conductive indium tin oxide (ITO)–coated glass slides (Bruker Daltonics, Bremen, Germany) for MALDI-MSI. All specimens were stored at − 80 °C until use.

### Amyloid staining and fluorescence imaging (FI)

For plaque visualization, sections were fixed sequentially in 95% ethanol (1 min), 50% ethanol in water (30 s), and water (1 min), followed by incubation with the optotracer Amytracker 520 (1:1000 in water; Ebba Biotech AB, Stockholm, Sweden) for 30 min at room temperature in the dark. Sections were rinsed briefly in water, desiccated, and stored at 4 °C until imaging. This LCO staining previously shown to co-localize with Aβ plaque- and tau tangle-specific antibodies [33, 34]. The Aβ plaques were identified based on size, staining positivity, and MSI Aβ signal positivity, and the NFTs were identified based on size, fibrillar, thread-like tangle structure, and staining positivity. Fluorescent imaging was performed on an automated widefield scanner (Axio Scan Z1, Zeiss, Oberkochen, Germany). Large multi-channel z-series tile scans were acquired using a Plan-Apochromat 20 × /0.8 DIC air objective and an ORCA monochrome camera (Hamamatsu, Shizuoka, Japan). Excitation and emission filters were matched to the used fluorophores, specifically Alexa Fluor 488 (LED 475 nm, 450–488/504–546 nm). Image processing of the FI images was done in QuPath v0.6.0 [35].

### Matrix deposition for MALDI-MSI

Fresh-frozen tissue sections mounted on ITO-coated glass slides were fixed in 100% ethanol for 60 s, followed by 70% ethanol for 30 s. Lipids were removed by immersion in Carnoy’s solution (ethanol/chloroform/acetic acid, 60/30/10 v/v/v) for 120 s, after which slides were sequentially washed in 100% ethanol (15 s), 0.2% trifluoroacetic acid in water (60 s), and 100% ethanol (15 s). For matrix deposition, a 60 mg/ml mixture of 2, 5-dihydroxybenzoic acid (DHB) and 2-hydroxy-5-methoxybenzoic acid (9:1) was prepared in acetonitrile/ddH₂O/trifluoroacetic acid (40/60/1 v/v/v) and applied using an M3 + sprayer (HTX Technologies, Carrboro, NC, USA).

### MALDI-MSI data acquisition and processing

Imaging experiments were performed on a rapifleX MALDI time-of-flight instrument (Bruker Daltonics) operated in reflector positive mode. Data were acquired at 20 µm spatial resolution across an m/z range of 2500–5000, with the laser operating at 5 kHz frequency, 52% power, detector gain set to 1.00 at 2280 V, ion source voltage of 19,989 V, reflector voltages of 20,832 V (Reflector 1), 8900 V (Reflector 2), and 8591 V (Reflector 3), and 100 shots accumulated per pixel. Prior to acquisition, the instrument was calibrated with red phosphorus. Tissue areas were selected and monitored using FlexControl 4.2 and FlexImaging 6.0 software (Bruker Daltonics). Data visualization and statistical analyses, including segmentation, were conducted in SCiLS Lab software (v2025a Pro, Bruker Daltonics). In SCiLS, data was baseline subtracted and TIC normalized. Identification of Aβ peptides was done by matching the experimental data against a macro-based curated mass list of all possible human Aβ peptide variants. Peaks corresponding to Aβ species were considered ‘detected’ if all of the following were true, (i) a clear signal was observed at the expected m/z in the spectrum, (ii) signal could be distinguished from baseline noise (SNR > 3), and (iii) peptide envelope for the given peptide was present. Tissue segmentation was performed in SCiLS Lab using bisecting k-means with a Euclidean distance metric and weak denoising, without spatial smoothing.

### Alignment of MALDI-MSI and fluorescence microscopy data for plaque analysis

Registration between the two modalities, MSI and fluorescence microscopy, was performed in SCiLS by aligning anatomical landmarks, tissue boundaries, and the Aβ MSI signal. The accuracy of the registration was assessed visually and is therefore limited to the optical resolution of the images (~ 10–20 µm). This level of precision was sufficient for comparing MSI cluster patterns with LCO fluorescence staining. No additional quantitative assessment of alignment was performed, as this study focused on overall plaque populations rather than intra-plaque features. At 20 µm resolution, this approach was adequate to verify the relevant pathological features. When single-plaque analysis was performed, the number of deposits included was limited to that in the subject with the fewest Aβ plaques (effectively rounded up to 300), and the analysis was performed in R (v4.4.1) Studio via random sampling to ensure reproducibility. This was done to avoid over-representation by individual patients.

### Immunoprecipitation and quantification of soluble Aβ protofibrils

Fresh frozen cerebral tissue (100 mg) was homogenized in (Tris)-buffered saline (TBS), pH 7.6 containing protease and phosphatase inhibitors (cOmplete™ Protease Inhibitor Cocktail and PhosSTOP™ Phosphatase Inhibitor Cocktail, Roche Diagnostics, Mannheim, Germany) in a 1:5 w/v ratio using a Potter-Elvehjelm homogenization tubes. After centrifugation (16,000 × g for 1 h at 4 °C), the supernatant, i.e., TBS fraction, was collected, aliquoted and stored at − 80 °C. Immunoprecipitation (IP) was performed using the KingFisher Apex System (Thermo Fisher Scientific, Waltham, MA, USA). TBS extracts were diluted 1:10 in IP buffer (Dulbecco’s PBS, 0.1% BSA, 0.5% Tween-20) supplemented with the Aβ protofibril selective antibody mAb158. M-280 Tosyl-activated Dynabeads (Invitrogen, Carlsbad, CA, USA) coupled with mouse-anti-mouse IgG2a (Pharmingen, San Diego, CA, USA) were added and allowed to bind to the mAb158-protofibril complexes. Beads were washed in IP buffer and immunoprecipitated Aβ protofibrils were eluted in 70% formic acid. Extracts were then neutralized 1:30 in a buffer consisting of equal volumes of Diluent 35 (Meso Scale Discovery, Rockville, MD, USA) and 1 M Trizma base and 0.5 M Na_2_HPO_4_. Samples were analyzed using V-PLEX Aβ Peptide Panel (4G8) Kit according to manufacturer’s instructions (Meso Scale Discovery). The plate was analyzed using a Mesoscale Sector Imager and levels of Aβx-38, Aβx-40 and Aβx-42 in samples were back calculated against standard curves.

### Statistical analysis

GraphPad Prism software version 9.5.0 (GraphPad Software, La Jolla, CA, USA) and R studio (v4.4.1) were used for statistical analysis. The normality of the data was assessed using the Shapiro-Wilk test. An unpaired t-test or Mann-Whitney test was used to compare the means or medians, respectively, between two groups. The non-parametric one-way ANOVA on ranks (Kruskal-Wallis) followed by Dunn’s test for multiple comparisons was used when comparing more than two groups. For single plaque correlation analysis, data were log-transformed followed by Pearson correlation.

## Results

### Morphological differences in Aβ and NFT pathology between DS and sAD

Aβ and neurofibrillary tangle (NFT) deposits are hallmark lesions of AD and related neurodegenerative disorders, yet they are far from uniform in structure or appearance. Previous neuropathological studies have shown that both Aβ and NFT aggregates vary in size, density, fibrillar architecture, and regional distribution, not only across AD subtypes but also between distinct proteopathies [36–38]. These variations likely reflect differences in aggregation kinetics, local microenvironments, and upstream genetic drivers. In familial forms of AD, pathogenic variants alter APP processing; in sAD, mechanisms are more heterogeneous and less well characterized. In DS, lifelong APP overexpression is expected to accelerate Aβ accumulation, but whether this also yields distinctive morphological signatures of Aβ has been less clear.

**Fig. 1:**
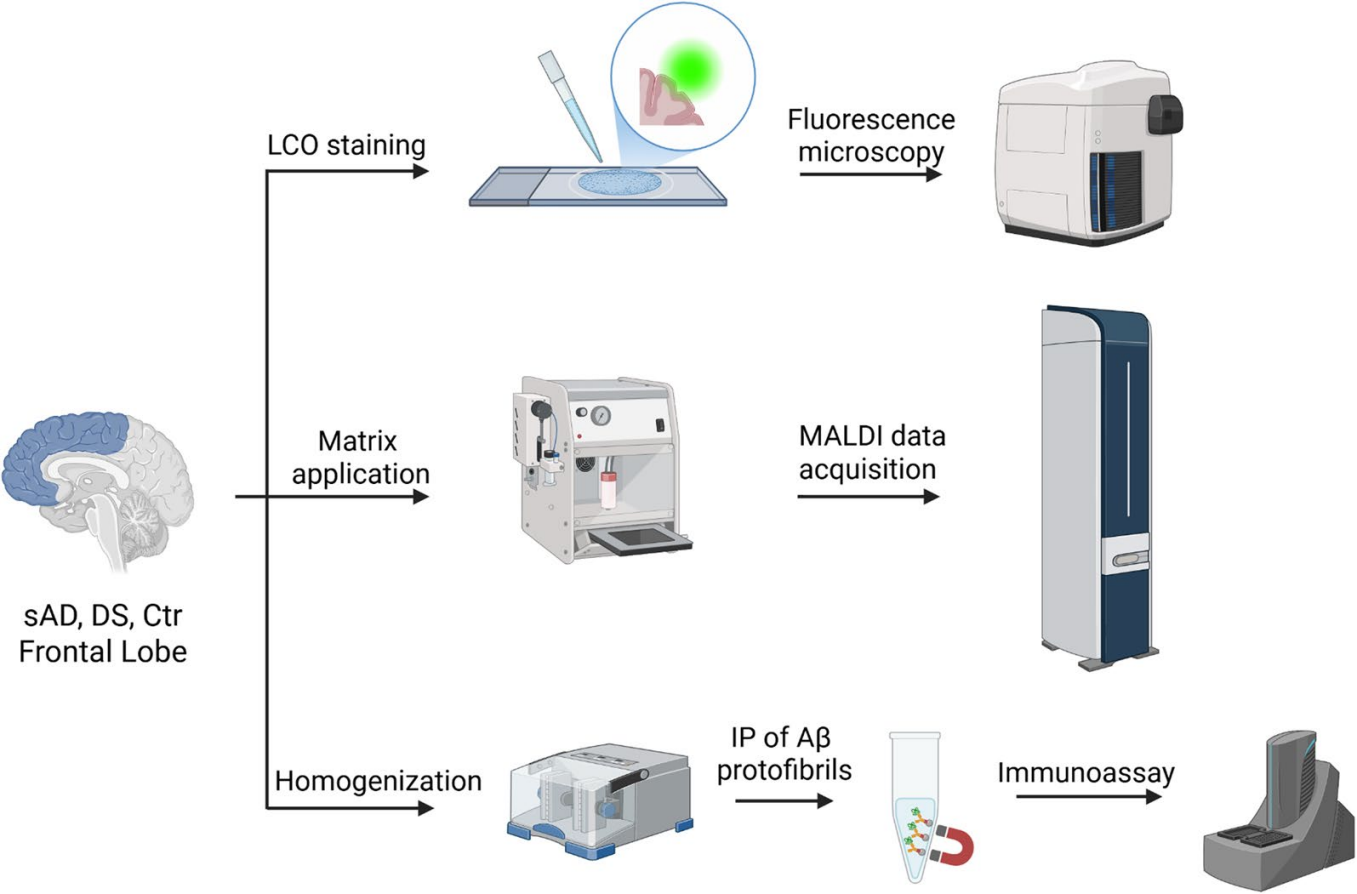
Multimodal Aβ analysis workflow. Schematic presentation of the workflow for the fluorescence imaging using luminescent amyloid probes (top), MALDI MSI analysis of Aβ peptide variants (middle) and IP-MSD analysis of Aβ protofibrils (bottom) in human brain tissue from sAD, DS and non-demented control subjects. Created with BioRender.com

**Fig. 2:**
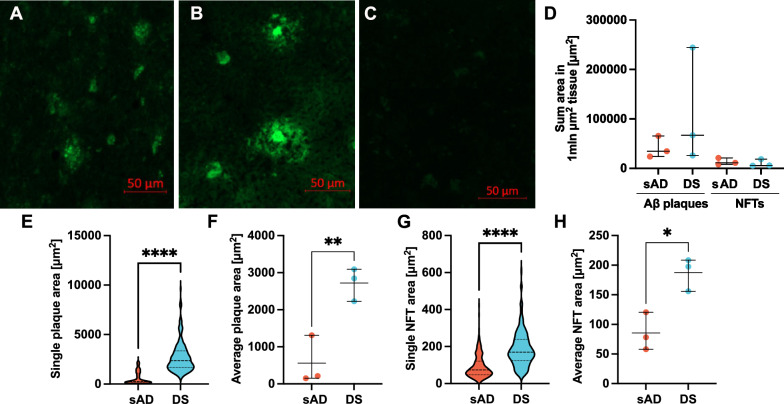
Quantification of Aβ plaques and NFTs in sAD and DS. Representative examples of fluorescence imaging of Aβ deposits and NFTs in human brain tissue from sAD (**a**) DS (**b**) and non-demented control (**c**). Comparison of plaque and tangle densities in 1mln µm^2^ tissue (**d**) between sAD (n = 3) and DS (n = 3) patients. Each symbol represents the total area of all deposits collected per patient. A Kruskal-Wallis test was conducted to compare differences among these groups, followed by Dunn’s post hoc test. Comparison of single (**e**,** g**) and average (**f**, **h**) areas of Aβ plaques (**e**–**f**) and NFTs (**g**–**h**) in brain tissues. Comparisons between two groups were performed using a Welch’s t-test for normally distributed data and a Mann-Whitney test for non-normal data. A *p*-value ≤ 0.05 was considered statistically significant

We therefore set out to systematically interrogate lesion morphology and link structural features to underlying chemical phenotypes in DS, sAD, and non-demented control brains. To do so, we combined high-sensitivity amyloid-targeted histology, reflector-mode Aβ peptide analysis, and electrochemiluminescence-based detection of Aβ and tau aggregates using luminescent conjugated oligothiophenes (LCOs) (Fig. 1) [24, 30]. Histological inspection revealed abundant Aβ plaques and NFTs in both sAD and DS (Fig. 2a–b), while tissues from non-demented controls were consistently negative for LCO and Aβ signal in MSI (Fig. 2c, Supplementary Fig. 1) in agreement with Braak staging. For each patient, Aβ plaques and NFTs were manually selected across the entire tissue section. Pathological features were included when their identity and boundaries could be unambiguously confirmed by their characteristic morphology and LCO staining patterns. All included structures met the same morphological inclusion criteria. Quantitative tissue density estimates revealed no significant differences in the number of Aβ plaques or NFTs per unit area between sAD and DS, suggesting comparable overall amyloid load in these groups (Fig. 2d). Next, we inspected the morphology of the aggregates by measuring the surface area of Aβ plaques and NFTs. This unbiased sampling revealed a striking size difference: both Aβ plaques and NFTs were larger in DS than in sAD (Fig. 2e–h). The distribution of individual Aβ plaques in DS patients was much higher than in sAD patients (Fig. 2e, *p*<0.0001), with the average Aβ plaque size approximately five times larger in DS patients than in sAD (Fig. 2f,  *p*= 0.009). The size distribution of tangles between sAD and patients largely overlapped (Fig. 2g, *p*< 0.0001), with the average tangle size being roughly double in DS patients compared with sAD (Fig. 2h, *p*= 0.01). Together, these observations suggest that the neuropathological landscape in DS is not merely accelerated but qualitatively distinct, with Aβ deposits co-existing alongside tau pathology.

### Aβ peptide repertoires revealed by reflector-mode MALDI imaging

Having established that Aβ lesions in DS differ morphologically from those in sAD, we next sought to examine their underlying molecular composition. Characterizing the biochemical heterogeneity of individual Aβ deposits has been challenging, as conventional extraction-based methods require large tissue amounts and often bias detection toward certain aggregation states [39]. MALDI-MSI addresses this challenge by enabling in situ analysis of Aβ pathology without prior extraction, preserving spatial context. However, systematic tissue characterization is still limited by technical constraints. Although linear TOF mode can offer higher intrinsic sensitivity for high-mass ions, in this study, we employed a newly optimized acquisition strategy in reflector mode, which enabled unambiguous assignment of Aβ peptides while maintaining sufficient sensitivity for tissue analysis.

**Fig. 3:**
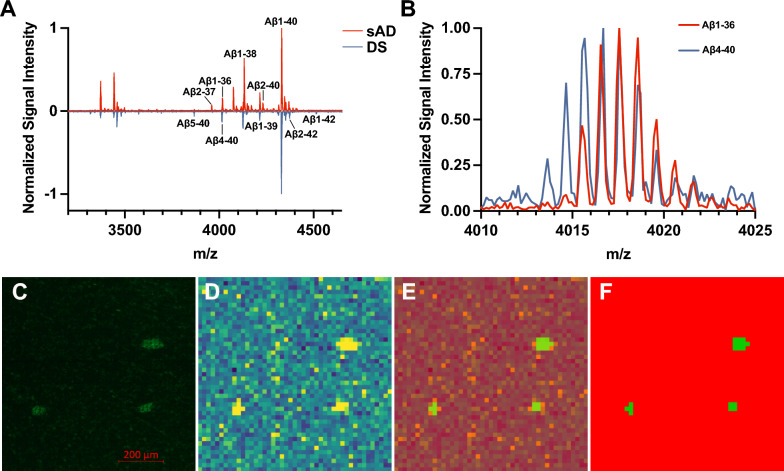
Reflector-mode MALDI imaging of Aβ isoforms in DS and sAD brain tissue. Normalized signal intensity spectra from representative sAD and DS patients, with annotation of few major Aβ peptides (**a**). Separation of overlapping isotope envelope patterns of Aβ1-36 and Aβ4-40 achieved using our novel reflector mode MSI approach (**b**). A representative brain tissue section from a DS patient is shown, illustrating (**c**) plaque localization via luminescent conjugated oligothiophene (LCO) staining, alongside MALDI-MSI-derived single-ion images of Aβ1-42 peptide (**d**), with corresponding 50% (**e**) and complete (**f**) segmentation map

Inspection of average spectra suggested that even on the tissue level the sAD subjects are dominated by more diverse Aβ truncations (Fig. 3a), as represented by the higher diversity of peptides in the lower mass range. At this stage we also confirmed that our newly developed method was able to separate individual Aβ peptides with overlapping isotope envelope patters (Fig. 3b). Following data acquisition, the resulting high-dimensional data were interrogated in an unbiased fashion using bisecting k-means clustering. This segmentation approach partitioned the tissue into clusters that corresponded closely to Aβ plaques, and the adjacent parenchyma region. Notably, the segmentation was data-driven and required no prior histological annotation; nevertheless, the overlap with LCO-stained plaques confirmed the accuracy of cluster identification (Fig. 3c–f).

**Fig. 4:**
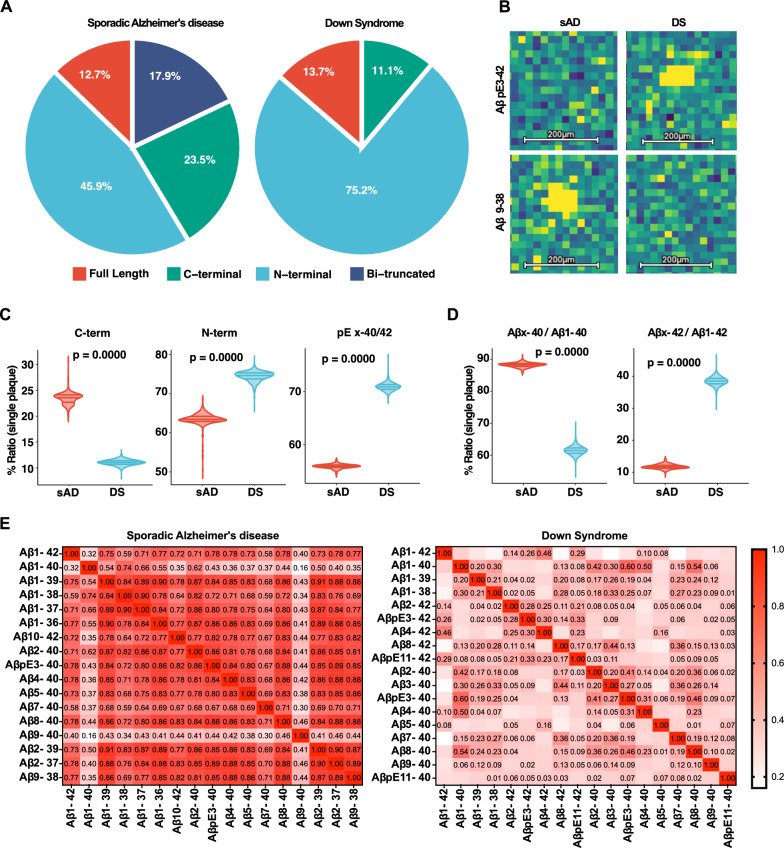
Aβ isoform distribution and single-plaque analysis in DS and sAD plaques. Average distribution of Aβ isoforms grouped into full-length, N-terminally truncated (Aβx-40/x-42), C-terminally truncated, and bi-terminally truncated peptides per patient (**a**). sAD plaques contained a twofold higher proportion of C-terminally truncated peptides compared to DS, while N-terminally truncated species were more abundant in DS. DS plaques were enriched in pyroglutamate-modified species such as AβpE3-42, largely absent in sAD, whereas sAD uniquely contained bi-terminally truncated species including Aβ2-37, Aβ2-39, and Aβ9-38 (**b**). Single-plaque analysis confirmed these differences are not driven by any individual patient (**c**). N-terminal truncations in sAD terminate predominantly at amino acid 40 (Aβx-40), whereas in DS predominantly at amino acid 42 (Aβx-42) (**d**). Pearson correlation matrices of Aβ isoforms within individual plaques reveal stronger inter-peptide co-occurrence in sAD than DS (**e**)

From these plaque-defined clusters, we extracted average ion spectra and peptide distributions, achieving comprehensive coverage of Aβ isoforms in both sAD and DS brains (Fig. 4a, Table 2). Among these, the canonical Aβ1-40 and Aβ1-42 peptides were consistently detected across all examined brain sections from both sAD and DS patients. In contrast, the presence and spatial distribution of other Aβ variants varied notably between cases. For instance, while pyroglutamate Aβ3-40 (AβpE3-40) was present in both sAD and DS patients, AβpE3-42 was uniquely detected in DS. Similarly, AβpE11-40 and AβpE11-42 were absent in sAD cases, but present in DS. These findings, delivered from segmentation-based MALDI-MSI analysis, suggest that Aβ plaques in DS harbor a more diverse and broader repertoire of Aβ isoforms, including Aβ2-42, AβpE3-42, Aβ3-40, Aβ4-42, Aβ8-42, AβpE11-40/42, which may be associated with plaque formation earlier as observed in this disorder.

**Table 2:** Aβ peptides list identified in DS and sAD

	Positions	Sequence	sAD	DS
C-truncated Aβ	1-42	DAEFRHDSGYEVHHQKLVFFAEDVGSNKGAIIGLMVGGWIA	x	x
	1-40	DAEFRHDSGYEVHHQKLVFFAEDVGSNKGAIIGLMVGGW	x	x
	1-39	DAEFRHDSGYEVHHQKLVFFAEDVGSNKGAIIGLMVGGV	x	x
	1-38	DAEFRHDSGYEVHHQKLVFFAEDVGSNKGAIIGLMVGG	x	x
	1-37	DAEFRHDSGYEVHHQKLVFFAEDVGSNKGAIIGLMVG	x	
	1-36	DAEFRHDSGYEVHHQKLVFFAEDVGSNKGAIIGLMV	x	
N-truncated Aβ	2-42	AEFRHDSGYEVHHQKLVFFAEDVGSNKGAIIGLMVGGWIA		x
	pE3-42	EFRHDSGYEVHHQKLVFFAEDVGSNKGAIIGLMVGGVVIA		x
	4-42	FRHDSGYEVHHQKLVFFAEDVGSNKGAIIGLMVGGWIA		x
	8-42	SGYEVHHQKLVFFAEDVGSNKGAIIGLMVGGVVIA		x
	10-42	YEVHHQKLVFFAEDVGSNKGAIIGLMVGGWIA	x	
	pE11-42	EVHHQKLVFFAEDVGSNKGAIIGLMVGGWIA		x
	2-40	AEFRHDSGYEVHHQKLVFFAEDVGSNKGAIIGLMVGGVV	x	x
	3-40	EFRHDSGYEVHHQKLVFFAEDVGSNKGAIIGLMVGGW		x
	pE3-40	EFRHDSGYEVHHQKLVFFAEDVGSNKGAIIGLMVGGW	x	x
	4-40	FRHDSGYEVHHQKLVFFAEDVGSNKGAIIGLMVGGW	x	x
	5-40	RHDSGYEVHHQKLVFFAEDVGSNKGAIIGLMVGGW	x	x
	7-40	DSGYEVHHQKLVFFAEDVGSNKGAIIGLMVGGW	x	x
	8-40	SGYEVHHQKLVFFAEDVGSNKGAIIGLMVGGW	x	x
	9-40	GYEVHHQKLVFFAEDVGSNKGAIIGLMVGGW	x	x
	pE11-40	EVHHQKLVFFAEDVGSNKGAIIGLMVGGW		x
Bi-truncated Aβ	2-39	AEFRHDSGYEVHHQKLVFFAEDVGSNKGAIIGLMVGGV	x	
	2-37	AEFRHDSGYEVHHQKLVFFAEDVGSNKGAIIGLMVG	x	
	9-38	GYEVHHQKLVFFAEDVGSNKGAIIGLMVGG	x	

Underlined letter E indicates N-terminal pyroglutamate modification

### Distinct truncation patterns of Aβ isoforms in DS and sAD

We next quantified the distribution of Aβ isoforms across plaque-segmented clusters. Aβ peptides arise from sequential cleavage of APP by β- and γ-secretases, typically producing full-length Aβ1-40 and Aβ1-42 as the dominant species. However, additional processing can occur: aminopeptidases can remove residues from the N-terminus (e.g., generating Aβ2-x, Aβ3-x, or Aβ11-x) [40, 41], while carboxypeptidases and alternative γ-secretase trimming can shorten the C-terminus by several residues (e.g., producing Aβ1-37, Aβ1-38, or Aβ1-39) [42]. When both N- and C-terminal processing events occur, bi-terminally truncated species are generated. Such modifications not only diversify the peptide pool but also strongly influence aggregation kinetics and plaque morphology [10]. Therefore, to capture these differences, peptides were grouped into four categories: full-length species, N-terminally truncated (Aβx-40/x-42), C-terminally truncated, and bi-terminally truncated peptides. Analysis of these peptide fractions across patients and individual plaques revealed striking differences between conditions (Fig. 4a). In sAD, average plaques contained a two-fold higher proportion of C-terminally truncated peptides than in DS (23.5% vs. 11.1%). On the other hand, the opposite trend was observed for N-terminally truncated species, which contributed roughly a quarter less of all signals (45.9% vs. 75.2%). Surprisingly, pyroglutamate-modified species were significantly less abundant in sAD than in DS, with forms such as AβpE3-42 largely absent (Fig. 4b, top). It is possible that this was due to the ApoE ε3/ε3 genotype of the sAD cases and the fact that these species become more prominent only as the Aβ plaques become older. Conversely, sAD plaques were enriched in bi-terminally truncated species, including Aβ2-37, Aβ2-39, and Aβ9-38 (Fig. 4b, bottom). This points to more complex, multi-step proteolytic cascades in sAD. These observations were confirmed at the single-plaque level, indicating that this separation is not driven by any single patient (Fig. 4c). Further quantification revealed that while in sAD the N-terminal truncations ended largely at amino acid 40 (Aβx-40), in DS they ended largely at amino acid 42 (Aβx-42) (Fig. 4d). Together, these results indicate that sAD and DS differ not only in the presence of specific Aβ peptides but also in the mechanisms of Aβ processing, with DS plaques shaped primarily by N-terminal modifications and sAD plaques characterized by broader truncation diversity.

### Peptide co-localization patterns and protofibrillar Aβ

To further interrogate the internal organization of plaques, we performed correlation analyses between different peptide signals within individual plaques across patients (Fig. 4e). This approach allowed us to assess the degree of spatial co-occurrence among different Aβ species, thereby providing insight into their aggregation relationships. The analysis revealed that different Aβ species exhibited significantly stronger inter-correlations in sAD compared to DS, where these associations were notably weaker.

To contextualize these spatial relationships with the soluble Aβ pool, we measured soluble aggregated Aβx-40 and Aβx-42 using electrochemiluminescence Meso Scale Discovery (MSD) assays. Levels of Aβ40, Aβ42, and Aβ40/42 did not differ substantially between DS and sAD, despite two outliers in each group (Supplementary Fig. 2), suggesting broadly comparable production and clearance of full-length species. Taken together, the integration of correlation analyses with protofibril measurements highlights that, despite similar soluble Aβ burdens, DS and sAD diverge markedly in the structural plaque assembly.

## Discussion

In this study, we investigated the spatial distribution, molecular composition, and aggregation patterns of Aβ peptides in DS and sAD. Using a combination of fluorescence histology, a reflector-mode MALDI-MSI approach, and electrochemiluminescence-based assays, we provide new insights into the differences in plaque and tangle morphology, peptide repertoires, truncation patterns, and spatial assembly of Aβ deposits between the two conditions.

Our data confirm and extend prior observations that although the abundance of Aβ and tau lesions is comparable between DS and sAD, the lesions in DS are significantly larger in size [20]. The presence of larger Aβ plaques and NFTs in DS suggests that Aβ and tau pathologies co-occur, leading to accelerated and more severe pathology relative to sporadic disease. While previous study has noted increased plaque size in DS [20], our findings provide new evidence that NFTs are similarly affected, underscoring a coordinated amplification of Aβ-tau interactions.

**Fig. 5:**
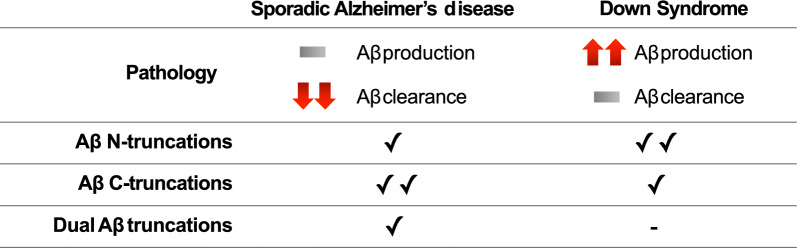
Schematic overview summarizing pathological features and key findings in sporadic Alzheimer’s disease and Down Syndrome

Beyond morphology, our plaque-resolved MALDI-MSI analysis uncovered fundamental differences in Aβ peptide composition (summarized in Fig. 5). DS plaques contained a broader and more diverse repertoire of Aβ species, including Aβ2-42, AβpE3-42, Aβ3-40, Aβ4-42, Aβ8-42, AβpE11-40/42, which were absent from sAD plaques. These truncated species are highly aggregation-prone and may contribute to the earlier onset and accelerated plaque development characteristic of DS. The detection of AβpE11 variants is particularly noteworthy given the overexpression of BACE2 in DS, a protease capable of generating N-terminal truncations at this position [43]. By contrast, sAD plaques displayed a narrower but chemically more heterogeneous pattern, characterized a set of unique bi-terminal truncations (i.e., Aβ2-37, Aβ2-39, Aβ9-38). The presence of these sAD-specific isoforms points to a more complex proteolytic environment, likely involving sequential action of amino- and carboxypeptidases. Interestingly, N-terminally truncated Aβ species such as pE3-42 and Aβ4-42, which have been reported as among the most abundant peptides in bulk cortical sAD samples [44], were less prominent in our MALDI-MSI analyses. In our dataset, these species were primarily detected in DS samples. This observation aligns with evidence that distinct truncated Aβ variants, including AβpE3-42, play prominent roles in human plaque pathology but may vary depending on plaque type and maturation [24], a pattern that may also be influenced by ApoE genotype, as all sAD cases in our study were ε3/ε3. Further studies, into Aβ peptide composition across ApoE genotypes, are however needed. Furthermore, these peptides are generally more abundant in bulk tissue, which integrates signals across many plaques, but also includes Aβ peptides of different aggregation state. Single-plaque MALDI-MSI captures plaque-specific signals and may therefore show comparatively lower levels. Together, these observations underscore the importance of single-plaque analyses for understanding plaque heterogeneity and suggest that some peptides abundant in bulk tissue may not be equally represented in individual plaques.

These differences became especially clear when we examined the relative amount of Aβ truncations. DS plaques were dominated by N-terminal truncations, with near-equal contributions from Aβ40- and Aβ42-derived species, suggesting a broadly acting aminopeptidase or alternative cleavage pathway. In contrast, sAD plaques contained a disproportionate enrichment of truncated Aβ40, while Aβ42 remained largely full-length. This asymmetry may indicate preferential processing of Aβ40 in sAD, whereas DS favors a non-selective mechanism that acts on both Aβ 40- and Aβ42-mers. Such divergence underscores that Aβ pathology in DS is not simply accelerated sAD, but follows distinct processing trajectories.

Our comparison of soluble Aβ protofibrillar pools through IP-MSD provided an additional layer of information. It indicated that soluble, yet aggregated forms of Aβ do not differ between the patients. Similarly to the previous studies [12, 19], the spatial density of Aβ and tau NFT was broadly similar between DS and sAD. This suggests that, at the given stage of disease, the overall production, clearance, and deposition of these species are balanced across the diseases. In light of the MSI data, these apparently similar pools masked deeper compositional and spatial differences, suggesting that, while the initial development of Aβ pathology in sAD and DS is driven by the same aggregation mechanism, the progression and maturation of the pathology are distinct and modulated by distinct processes. In both diseases, we observed relative outliers. The highest values observed for Aβx-40 in the sAD and DS groups corresponded to female individual cases with particularly extensive Aβ pathology (sAD2 and DS2). Other female subjects in both groups exhibited lower Aβx-40 levels, suggesting that sex is unlikely to contribute to the elevated Aβ levels observed in the current cohort. Instead, it is likely that the elevated levels of Aβx-40 and the ratio Aβx-40/Aβx-42 are caused by the high content of Aβ-covered vessels in the homogenate.

It is also worth noting that samples from non-demented controls exhibit only slightly lower protofibrillar Aβ42 levels as compared to sAD and DS cases (Supplementary Fig. 2), despite the absence of visible plaques and lack of quantifiable Aβ signal in MSI (Fig. 2 and Supplementary Fig. 1). Therefore, this likely reflects the presence of soluble or oligomeric Aβ species that are not organized into detectable aggregates. LCO used in this study primarily stains β-sheet–rich, aggregated peptides, so these soluble protofibrils would not be visualized by this dye. This observation is consistent with previous studies showing that protofibrillar Aβ can exist in tissue even in the absence of overt plaque deposition [45, 46]. Alternatively, the Aβ levels might come from vascular Aβ deposition (although not observed in the tissues analyzed with IHC and MSI).

In this study, we analyzed Aβ and NFT pathology only in patients with the APOE ε3/ε3 genotype. Although selective, this choice was based on the fact that APOE ε4 allele can directly influence the Aβ and NFT deposition, potentially introducing a confounding factor in delineating the Aβ processing machinery and pathology development. By limiting the cohort to ε3/ε3 individuals, we aimed to reduce variability attributable to APOE genotype and better isolate other pathological mechanisms. It should be noted that this study included a small number of participants (n = 3 per group) and was conducted as a pilot investigation using rare post-mortem brain tissue from DS patients. While our results provide valuable insights into plaque- and NFT-level pathology, the limited sample size constrains statistical power and generalizability, and observed trends should therefore be interpreted with caution. Larger cohorts and independent validation will be necessary to confirm these findings and establish their broader relevance to sAD and DS neuropathology. However, our single-plaque analyses still highlight substantial heterogeneity in Aβ composition both within and between individuals, as well as across disease groups, emphasizing that Aβ pathology should not be viewed solely through aggregate measures or pathology scores. Instead, our data underscore the molecular complexity of plaque-associated Aβ peptides and suggest that disease phenotypes are shaped not only by pathological burden but also by qualitative differences in peptide composition.

In summary, our study demonstrates that DS and sAD differ across several dimensions, including Aβ plaque and NFT morphology, peptide repertoires, truncation patterns, and spatial assembly principles. Despite the small cohort size, the analyses demonstrated robust plaque-level findings across all participants, capturing variability between individual plaques and between patients, while still retaining clear differences between disease groups. DS pathology, marked by continuous APP-driven overproduction, does not appear to influence Aβ protofibril balance as compared to sAD. Instead, the dominant discriminating factor is the packaging of Aβ deposits and dominant generation of N-terminal rather than C-terminal truncations. By contrast, sAD is defined by the emergence of bi-terminal truncations and disproportionate processing of Aβ42. Together, these distinctions underscore that DS represents not simply an accelerated form of sAD, but a distinct variant of Aβ pathology shaped by unique genetic and proteolytic mechanisms.

## Supplementary Information

Supplementary material 1 (PDF)

## Data Availability

The datasets generated during the current study are available from the corresponding author upon reasonable request.
