# Supplementary material for: MALDI mass spectrometry imaging (MSI) reveals molecular and structural heterogeneity of amyloid-β in sporadic Alzheimer’s disease and Down syndrome
Source: Acta Neuropathol Commun. 2026 Apr 2;14:87. doi: 10.1186/s40478-026-02280-4 (PMC13063766; doi:10.1186/s40478-026-02280-4)
Supplement: Supplementary file 1 — Supplementary material 1 (PDF) [file 40478_2026_2280_MOESM1_ESM.pdf]

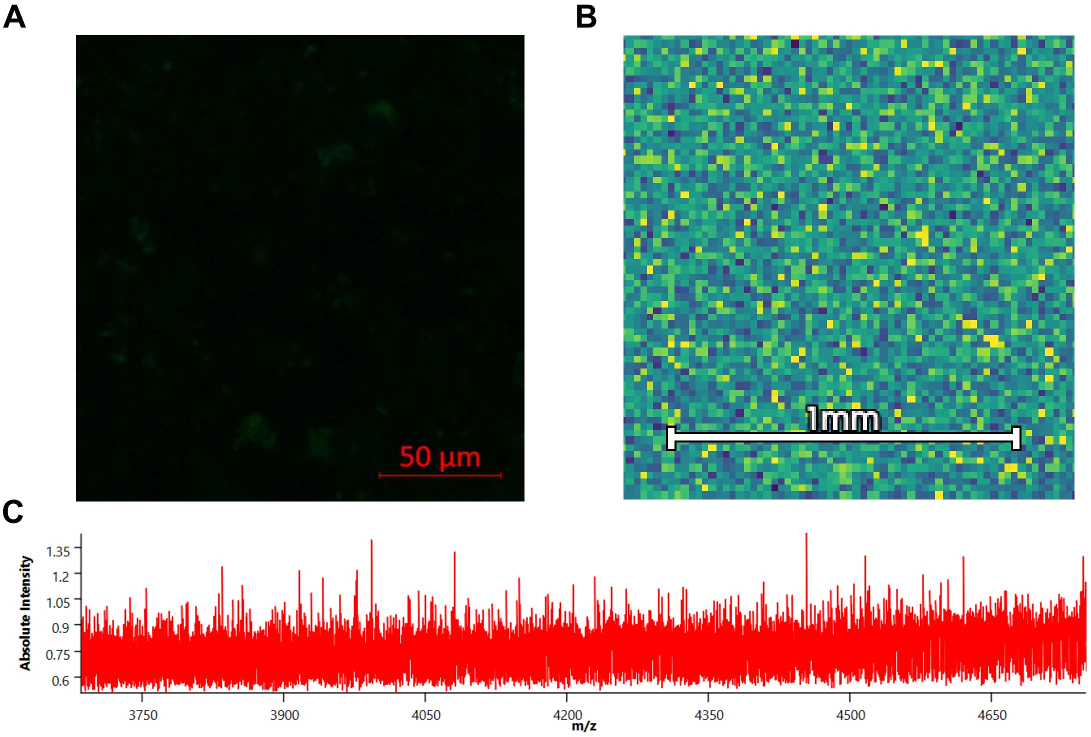

Supplementary Fig. 1. Example spectra from a non-demented control patient. Similar to fluorescent imaging data for non-demented control patients (a, and Fig 2c), inspection of MSI imaging data revealed no plaque-like features (b). Inspection of the spectra corresponding to the analyzed tissues revealed no quantifiable Aβ peptide signal (c).

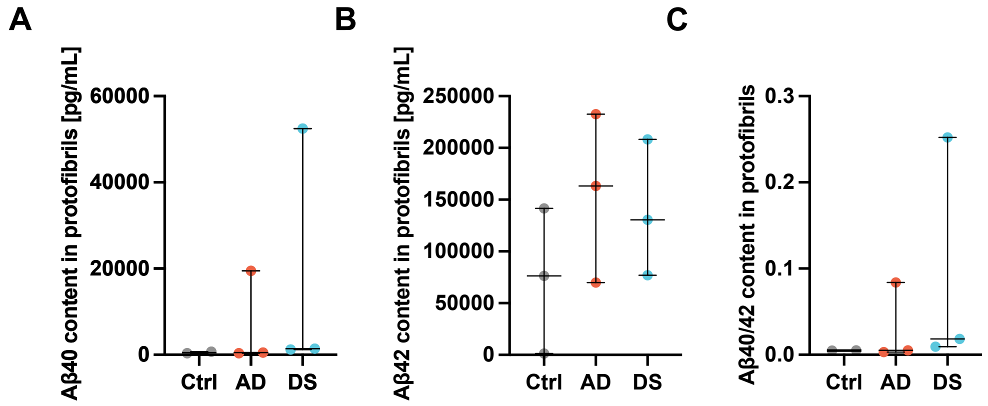

Supplementary Fig. 2. Comparison of Aβ protofibril levels. The comparison of Aβ40 (a), Aβ42 (b) and Aβ40/42 (c) contents in protofibrils between DS (n=3), sAD (n=3) and non-demented control (n=3) patients. One sample from a non-demented control had Aβ40 levels below the lower limit of quantification and was therefore excluded from the analysis. The plots display medians with interquartile ranges.
